# Microwave ablation versus parathyroidectomy for the treatment of primary hyperparathyroidism: a cohort study

**DOI:** 10.1007/s00330-022-08759-7

**Published:** 2022-04-06

**Authors:** Ying Wei, Zhen-long Zhao, Xiao-jing Cao, Li-li Peng, Yan Li, Jie Wu, Ming-an Yu

**Affiliations:** Department of Interventional Medicine, China-Japan Friendship Hospital, No. 2 Ying-hua-yuan East Street, Chao-yang district, Beijing, 100029 China

**Keywords:** Microwave radiation, Parathyroidectomy, Primary hyperparathyroidism, Parathyroid hormone

## Abstract

**Objective:**

To compare the clinical outcomes between microwave ablation (MWA) and parathyroidectomy (PTX) for the treatment of primary hyperparathyroidism (pHPT).

**Materials and methods:**

This retrospective study enrolled 212 patients with pHPT treated by either MWA (MWA group) or PTX (PTX group) from January 2015 to October 2020. The baseline data were balanced through propensity score matching. Clinical cure was evaluated by the Kaplan-Meier method and compared between the MWA and PTX groups. The risk factors related to persistent or recurrent pHPT were screening out using a Cox proportional hazards regression model.

**Results:**

After propensity score matching, a total of 174 patients were enrolled in the present study, with 87 patients in each group. During the follow-up period (median, 28.5 months), there were no differences between the two groups regarding the clinical cure (hazard ratio, 1.71; 95% confidence interval: 0.81-3.62; *p* = .155), persistent pHPT rate (13.8% vs. 10.3%, *p* = .643), recurrent pHPT rate (6.9% vs. 3.4%, *p* = .496), or major complications (6.9% vs. 3.4%, *p* = .496). MWA resulted in a shorter procedure time (30 min vs. 60 min), smaller incision length (0.1 cm vs. 7 cm) and slightly higher costs (25745 CNY vs. 24111 CNY) (all *p* < .001). High levels of preoperative intact parathyroid hormone (*p* = .01) and multiple pHPT nodules (*p* < .001) were independent risk factors for recurrent and persistent pHPT in the two groups.

**Conclusion:**

MWA and PTX have comparable clinical outcomes for pHPT. MWA has a shorter procedure time and smaller incision length.

**Key Points:**

*• There were no differences in terms of clinical cure, persistent pHPT, recurrent pHPT, or major complications between MWA and PTX in the treatment of pHPT.*

*• MWA is minimally invasive and results in a shorter procedure time.*

*• Multiple nodules and high levels of iPTH were the independent risk factors for recurrent and persistent pHPT.*

## Introduction

Primary hyperparathyroidism (pHPT) is the third most common endocrine disease, and is characterized as a group of mineral metabolism disorders [1, 2]. The global yearly incidence of pHPT is estimated to be between 1 and 4 cases per 1000 people and shows an obvious sex bias (female:male = 3–4:1) [3, 4]. Abnormally elevated parathyroid hormone (PTH) and hypercalcemia might cause a series of clinical symptoms, such as osteoporosis, fracture, nephrolithiasis, reduced renal function, impaired neurocognitive features, and even hypercalcemic crisis [1, 4]. In recent years, with the amelioration of socio-economic life conditions, more and more people begin to pay attention to routine physical examination and, as a result, the detection rate of asymptomatic pHPT has increased. It has been observed that 20–30% of asymptomatic pHPT could progress to symptomatic pHPT [5, 6].

Current treatment guidelines recommend parathyroidectomy (PTX) as the standard treatment for patients with pHPT [5, 7]. In recent decades, there have been several reports on minimally invasive treatments of pHPT, such as ethanol ablation [8], laser ablation [9], radiofrequency ablation [10], and high-intensity focused ultrasound [11]. As one of the most recent and exciting advances among thermal ablation techniques, microwave ablation (MWA) has been used for the treatment of pHPT [12–15]. Most studies have shown that MWA could effectively inactivate pHPT nodules and normalize serum PTH, calcium, and phosphorus [11–15]. A preliminary study comparing MWA with PTX showed that the two modalities achieved similar cure rates in pHPT [16]. However, limited by a small sample size and a short follow-up period, definitive evidence for treatment effectiveness is not well described. Therefore, in the present study, a comparative study between MWA and PTX was further conducted with a larger sample size and a relatively longer follow-up period; possible risk factors for recurrent and persistent pHPT were first analyzed to obtain more definite results about MWA for pHPT and obtain more technical details for preventing operative failure.

## Materials and methods

### Study design and patients

This is a retrospective cohort study. The electronic clinical records system in China-Japan Friendship Hospital was searched to retrieve data from all consecutive patients who underwent MWA or PTX for pHPT between January 2015 and October 2020. Only patients with sporadic pHPT who had complete clinical data were enrolled. Patients with misdiagnosed sHPT, familial pHPT, or parathyroid carcinoma were excluded. The study protocol was approved by the Human Ethics Review Committee of our hospital. All patients provided written informed consent for treatment, and informed consent for participation in the current study was waived because no identifying individual information would be presented. In our clinical practice, the benefits and risks of both treatment modalities will be explained to patients before making decisions. Patients could choose either PTX or MWA, and those who refused or were ineligible for PTX but met the criteria of MWA were enrolled in the MWA group.

### Imaging diagnosis

All enrolled patients underwent ultrasound (US) and ^99^mTc sestamibi (MIBI) examinations before treatment. The US examination was performed by using a LOGIQ E9 scanner (GE Healthcare with a 6–15-MHz linear probe. MIBI was conducted by a SymbiaT2 scanner (Siemens Healthineers) [5,17]. The diagnostic characteristics of pHPT nodules on US included (1) enlarged hypoechoic parathyroid glands with clearly defined margins and (2) no suspicion of lymph node metastasis. The MIBI characteristic of pHPT nodules was radioactive concentration in early and delayed phases.

### The procedure of microwave ablation

**Fig. 1:**
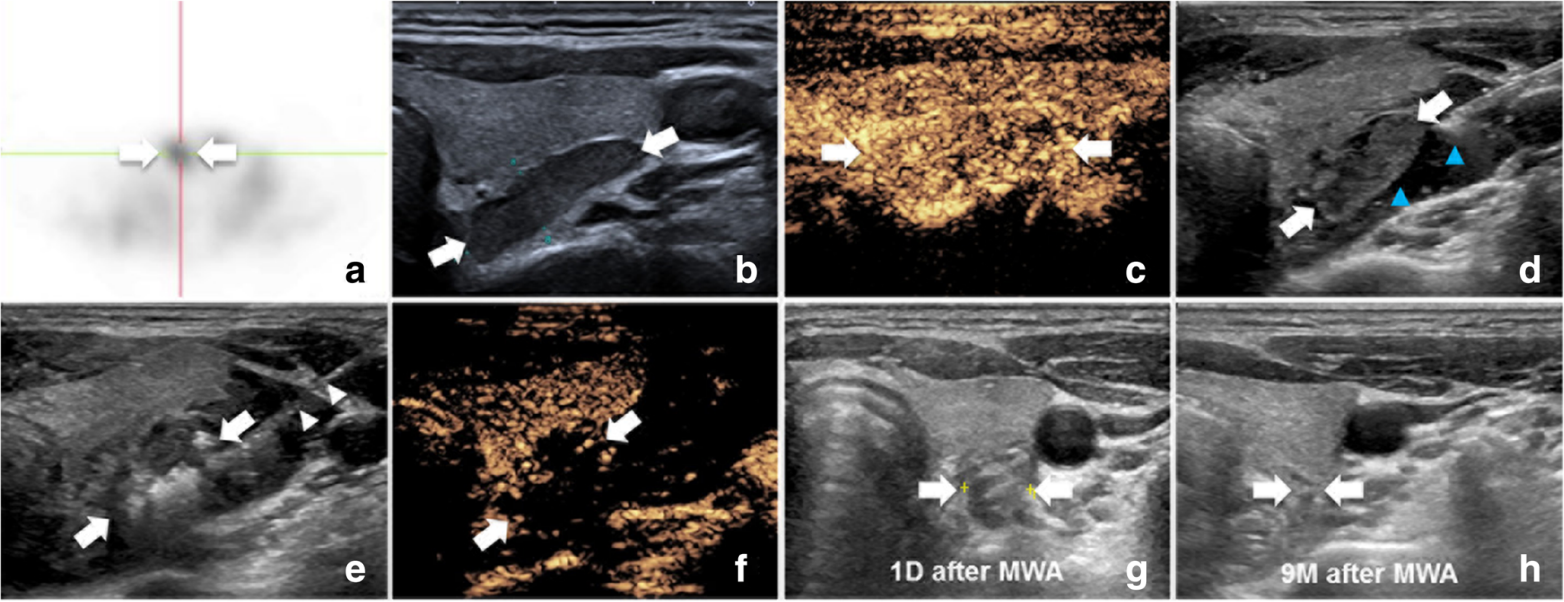
Images show MWA procedure of pHPT nodule. Ablation procedure of a pHPT nodule in a 40-year-old female. **a** There was radioactive concentration in pHPT nodule (white arrows) on MIBI. **b** Ultrasound showed a hypoechoic pHPT nodule (white arrows) behind superior right lobe of thyroid. **c** CEUS showed a hyperenhanced pHPT nodule (white arrows) in arterial phase. **d** Establishment of hydrodissection (blue arrowheads) around pHPT nodule (white arrows). **e** Ablation procedure: hyperechoic zone around antenna (white arrowheads) was emerging inside the pHPT nodule (white arrows). **f** CEUS showed a nonenhancement zone covering the pHPT nodule (white arrows). **g** One day after MWA, the ablation zone (2.0cm × 0.9cm). **h** Nine months after MWA, the ablation zone was absorbed. When performing MWA, the operator was on the head side of the patient and the side of ultrasound image was inverse. pHPT = primary hyperparathyroidism, CEUS = contrast-enhanced US, MIBI = ^99^mTc-sestamibi, MWA = microwave ablation, D = day, M = month

Patients were placed in a supine position with the neck extended. The ablation site was routinely sterilized and draped with sterile towels. First, 0.9% normal saline (NS) through an 18-gauge percutaneous transhepatic cholangiography needle was injected into the area around the parathyroid nodule to provide hydrodissection (at least 0.5 cm in distance between the parathyroid and adjacent critical structures). Then, a mixture of 1% lidocaine and NS (1:3) was administered near the periparathyroid capsule for local anesthesia. A cooled MWA antenna with a 3-mm active tip (Intelligent Basic Type Microwave Tumor Ablation System, KY-2000, Kangyou Medical; or Nanjing ECO Microwave System) was inserted into the target pHPT nodule under US guidance; a multipoint ablation technique was adopted for the ablation procedure. The power was 30 W, and the radiation time was 15–20 s at each point. The therapy was terminated when the hyperechoic zone covered the entire nodule. Contrast-enhanced US was performed 3–5 min later to assess the efficacy. Complete ablation was achieved if a nonenhanced zone covered the ablated nodule on contrast-enhanced US; if there was enhancement inside the ablation zone, additional ablation was performed immediately. Representative images of the ablation process are shown in Fig. 1. For patients with bilateral pHPT nodules, contralateral side ablation was performed only if vocal cord movement was normal on US and no voice change was noted after one side was ablated.

Multipoint ablation referred to the following: in each point ablation, a fixed applicator ablation was performed, then the antenna tip was moved to the next point guided by ultrasound and the next ablation was performed until the overlap ablation zone covered the entire pHPT lesion.

### The procedure of parathyroidectomy

The patient was placed in a supine position, with a shoulder pillow, with the head tilted back. After successful general anesthesia, the operation site was routinely sterilized and draped with sterile towels. Two transverse fingers were taken from the sternal notch, and the transverse neck incision along the dermatoglyphic direction was approximately 5–8 cm. The skin, subcutaneous tissue, and muscle were cut layer by layer for full dissociation, and bilateral sternohyoideus and sternothyreoideus muscles were separated to visualize the parathyroid gland and to perform PTX. The ipsilateral recurrent laryngeal nerve (RLN) was explored by nerve monitoring to protect it intraoperatively. Hemostasis was fully achieved, and the incision was sutured layer by layer. All patients underwent bilateral neck exploration with an attempt to identify all abnormal parathyroid glands.

### Follow-up

The follow-up was performed at 1 month and 3 months after treatment and then at 6-month intervals thereafter. The clinical data collected included blood biochemistry analyses (i.e., serum iPTH, phosphorus, calcium, alkaline phosphatase, vitamin D level, and estimated glomerular filtration rate), hospitalization time, procedure time, incision length, costs, and US examination.

The nodule volume in the MWA group was calculated according to the sphere volume formula: Volume = πabc/6 (a, the largest diameter; b and c are the other two perpendicular diameters). The volume reduction rate (VRR) was formulated as follows: VRR = 100 × (initial volume − final volume)/initial volume.

Referring to the guidelines for the management of pHPT, clinical cure was defined as reestablishment of normal values of serum calcium and iPTH throughout the entire follow-up period [5, 7]. Persistent pHPT referred to a failure to achieve normal serum calcium or iPTH within 6 months, and recurrent pHPT referred to the recurrence of hypercalcemia and/or an elevated iPTH level 6 months after treatment [17]. Operative failure was defined as a failure to normalize serum intact parathyroid hormone (iPTH) and/or calcium levels at least 6 months [18]. Complications during the treatment procedure and in the follow-up period were recorded.

### Statistical analysis

Statistical analysis was performed by using Stata 13.0 (StataCorp LLC) and R Studio 4.0.2 (R Foundation for Statistical Computing). Continuous data are presented as the mean ± standard deviation or the median and 25–75% interquartile range (IQR) if the data did not fit a normal distribution. Propensity score matching was used to control for baseline imbalances between the two groups. The propensity to undergo MWA versus PTX was estimated by using a logistic regression model based on age, sex, preoperative blood biochemical results, pHPT-related laboratory tests, nodule diameter, and nodule location. The matching algorithm was 1:1 genetic matching with no replacement, which automatically establishes a balance to determine the optimal weight for each covariable within the matching algorithm. The Wilcoxon rank sum test or *t* test was used for continuous variables, and the chi-square test or Fisher exact test was used for categorical variables as appropriate.

Clinical cure was assessed by using the Kaplan-Meier method and was compared by using the log-rank test. Risk factors associated with persistent or recurrent pHPT were analyzed by using a Cox proportional hazards regression model. Variables with *p* < .20 in univariable analyses were included in the multivariable models. All tests were two sided, with *p* < .05 considered to indicate a statistically significant difference.

## Results

### Patients

**Fig. 2:**
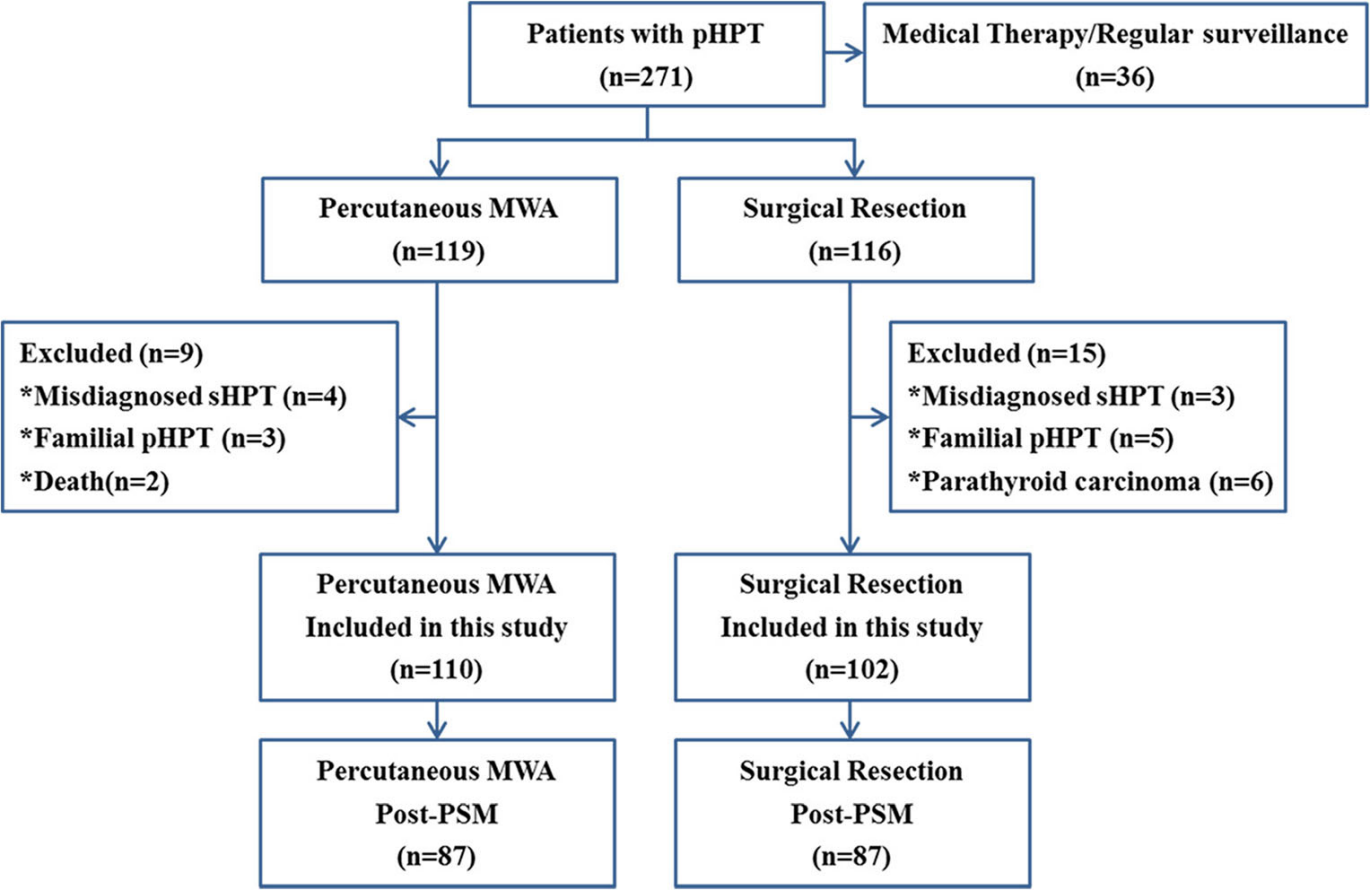
Patient flowchart. PTX = parathyroidectomy, MWA = microwave ablation, PSM = propensity score matching, pHPT = primary hyperparathyroidism

A total of 271 consecutive patients with pHPT were reviewed in the present study; 212 patients met the inclusion criteria. Among the 212 patients, 110 (51.9%) underwent MWA, and 102 (48.1%) underwent PTX. After matching, a total of 174 patients were ultimately enrolled, with 87 in the MWA group and 87 in the PTX group (Fig. 2). A good balance was achieved for most baseline parameters, except nodule diameter (MWA vs. PTX: 1.4 cm vs. 1.7 cm; *p* = .017) (Table 1).

**Table 1:** Baseline participant characteristics

Parameter	Unmatched Cohort		*p*	Matched Cohort		*p*
	MWA(110)	PTX (102)		MWA(87)	PTX (87)	
Female (*n*)	71	67	0.886	57	58	1.000
Age (years)	56.6 ± 16.2	54.2 ± 14.5	0.264	55.1 ± 16.8	53.5 ± 14.4	0.502
BMI (kg/m^2^)	24.5 ± 2.9	24.1 ± 3.6	0.382	24.4 ± 3.0	24.1 ± 3.6	0.629
No. of symptomatic pHPT	62	59	0.759	51	46	0.542
Serum iPTH (pg/mL)	163.6 (97.3–547.9)	220.3 (104.8–947.2)	0.002	201.2 (111.3–610.0)	190.0 (104.3–575.0)	0.953
Serum calcium (mmol/L)	2.8 ± 0.3	2.9 ± 0.3	0.086	2.8 ± 0.3	2.8 ± 0.3	0.726
Serum phosphorus (mmol/L)	0.9 ± 0.2	0.8 ± 0.2	0.723	0.8 ± 0.2	0.9 ± 0.2	0.324
ALP (U/L) regenerative growth	78 (50–184)	106 (62–282)	< 0.001	79 (58–178)	101 (62–169)	0.057
Vitamin D (nmol/L)	32.0 (14.9–80.7)	32.6 (14.0–70.2)	0.466	31.2 (14.2–75.7)	32.9 (14.6–73.6)	0.925
Ureanitrogen (mmol/L)	4.5 (2.4–7.1)	4.8 (2.8–8.6)	0.167	4.4 (2.3–6.9)	4.8 (2.9–8.4)	0.097
Creatinine (umol/L)	63.9 (44.1–108.7)	67.2 (43.8–135.2)	0.227	64.0 (43.7–105.9)	67.2 (44.1–125.8)	0.703
eGFR (mL/min/1.73 m^2^)	95.3 (44.5–127.9)	96.2 (43.6–128.5)	0.564	95.2 (49.0–128.2)	97.9 (49.2–129.4)	0.911
ALB(g/L)	42.8 ± 4.5	43.8 ± 3.6	0.222	42.9 ± 4.6	43.7 ± 3.9	0.209
HGB(g/dL)	131.5 ± 16.7	132.2 ± 16.2	0.882	131.5 ± 17.6	132.7 ± 15.8	0.624
Nodule diameter (cm)	1.4 (0.6–3.3)	2.0 (1.0–3.8)	< 0.001	1.4 (0.6–3.7)	1.7 (1.0–3.5)	**0.017**
Nodule Volume (mL)	0.5 (0.05–4.0)	0.7 (0.1–5.3)	0.005	0.5 (0.08–5.3)	0.5 (0.1–4.4)	0.282
No. of nodules			0.195			1.000
Single nodule	98	90	–	79	80	–
Two nodules	10	11	–	6	6	–
Three nodules	2	1	–	2	1	–
Nodule location			0.224			0.154
Superior left	23	23	–	17	20	–
Inferior left	49	36	–	39	32	–
Superior right	19	19	–	14	14	–
Inferior right	32	35	–	26	28	–
Ectopic	1	2	–	1	1	–

*MWA* microwave ablation, *PTX* parathyroidectomy, *BMI* body mass index, *iPTH* intact parathyroid hormone, *ALP* alkaline phosphatase, *ALB* albumin, *HGB* hemoglobin

Bold indicates *p* < 0.05. Normal reference: iPTH (12–88 pg/mL), serum calcium (2.00–2.75 mmol/L), serum phosphorus (0.81–1.78 mmol/L), ALP (40–150 U/L), vitamin D (75–250 mmol/L), ureanitrogen (2.78–7.85 mmol/L), creatinine (35–106 umol/L), ALB (35.0–55.0 g/L), HGB (115–150 g/dL)

### Intraoperative and postoperative outcomes

In the MWA group, 87 patients (97 pHPT nodules) received 91 treatment sessions. Among the 87 cases, 83 were treated with one session, and 4 were treated with two sessions. Nodular enhancement was observed in the ablation zone in 3 patients by immediate CEUS and complete ablation was achieved by additional ablation during one procedure. The reasons for performing the second session were varied; two patients received additional ablation due to missing ablation in the first session; the MWA antenna missed targeting the small pHPT nodule, which was mainly attributed to interference of hypoechoic hydrodissection with the clear display of the pHPT nodule on US. The third elderly patient with a large pHPT nodule (nodule diameter = 4.7 cm) underwent an additional complete ablation 3 months after the first session—a strategy of fractional ablation was planned mainly in consideration of safety. The fourth patient received additional ablation 1 month after the first session because the hematoma during the first procedure seriously interfered with the clear display of the second pHPT nodule. Among the 87 patients (96 pHPT nodules) in the PTX group, 86 underwent one surgery; another patient underwent two procedures due to another posterior sternal ectopic pHPT nodule. There were no differences in the treatment procedure between the two groups (*p* = .368).

The procedure time in the MWA group was shorter than that in the PTX group (MWA vs. PTX, median 30 min vs. 60 min, *p* < .001). The median incision length was 7 cm for the PTX group, but there was only one 1-mm pinhole in the MWA group (*p* < .001). The hospitalization cost was slightly higher in the MWA group (MWA vs. PTX, 25745 CNY vs. 24111 CNY, *p* = .026). There was no difference in hospitalization time between the two groups (MWA vs. PTX, 6 days vs. 5 days, *p* = .702) (Table 2).

**Table 2:** Comparison of intraoperative and postoperative outcomes between the MWA and PTX groups after propensity-score matching

Parameter	MWA (*n* = 87)	PTX (*n* = 87)	*p*
Procedure time (min)	30 (24–42)	60 (28–126)	< 0.001
Hospitalization time (days)	6 (2–16)	5 (3–19)	0.702
Incision length (cm)	0.1 (0.1–0.2)	7 (1–8)	< 0.001
Cost (CNY)	25745 (19745–37826)	24111 (14002–35746)	0.026
Persistent PHPT	12 (13.8)	9 (10.3)	0.643
Recurrent PHPT	6 (6.9)	3 (3.4)	0.496
Major complication	6 (6.9)	3 (3.4)	0.496
Voice change	6 (6.9)	2 (2.3)	0.278
Dyspnea caused by hematoma	0	1	1.000
Transient hypoparathyroidism	29 (33.3)	54 (62.1)	< 0.001
Transient hypocalcemia	4 (4.6)	16 (18.4)	0.008
Duration of transient hypoparathyroidism (days)	1 (1–18.5)	1 (1–30)	0.196
Duration of transient hypocalcemia (days)	1 (1–5.5)	1 (1–13.9)	0.682

Data are means ± standard deviations or medians with interquartile ranges in parentheses for continuous variables and are numbers of patients with percentages in parentheses for categorical variables

*MWA* microwave ablation, *PTX* parathyroidectomy, *pHPT* primary hyperparathyroidism

### Therapeutic efficacy

**Fig. 3:**
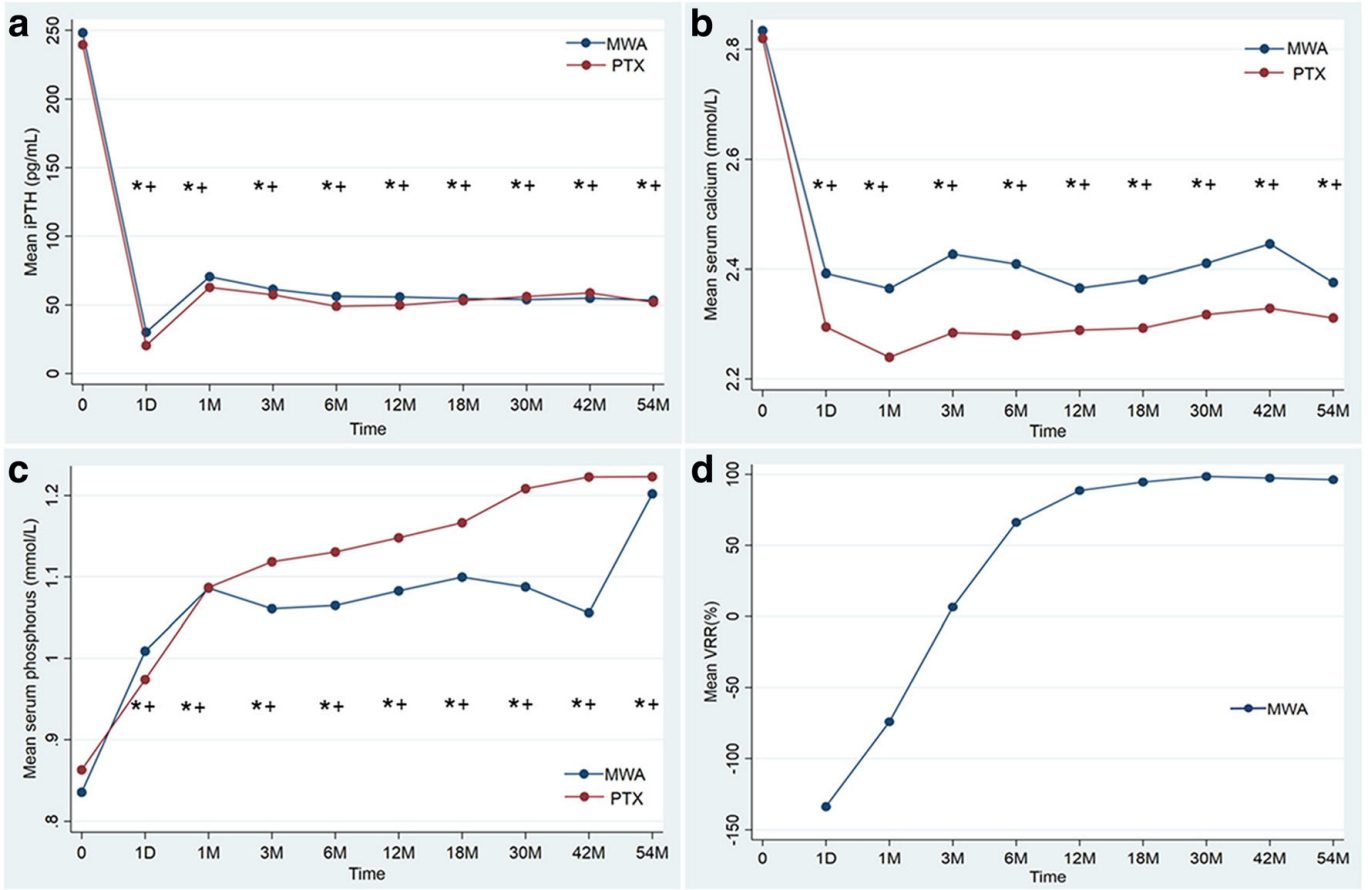
**a**–**c** Changes of serum biochemical parameters in MWA and PTX groups. **d** Changes of VRR of pHPT nodules in MWA group. iPTH = intact parathyroid hormone, M = month, D = day, VRR = volume reduction rate, PTX = parathyroidectomy, MWA = microwave ablation. *p < .05 vs. before operation. ^+^*p* > .05 MWA vs. PTX

Compared to pretreatment, the serum iPTH, calcium, and phosphorus levels were significantly improved 6 months after treatment in the two groups (in the MWA group: iPTH, 201.2 pg/mL vs. 43.6 pg/mL; calcium, 2.8 ± 0.3 mmol/L vs. 2.3 ± 0.2 mmol/L; phosphorus, 0.8 ± 0.2 mmol/L vs. 1.1 ± 0.2 mmol/L, respectively; in the PTX group: iPTH, 190.0 pg/mL vs. 50.9 pg/mL; calcium, 2.8 ± 0.3 mmol/L vs. 2.4 ± 0.2 mmol/L; phosphorus, 0.9 ± 0.2 mmol/L vs. 1.1 ± 0.2 mmol/L, respectively; all *p* < .05). There were no significant differences in the values of serum iPTH, calcium, or phosphorus between the two groups before treatment and during the follow-up period (all *p* > .05) (Fig. 3a–c). The volume of the ablation zone began to significantly decrease 1–6 months after MWA, and the mean VRR was 88.5% at 12 months (Fig. 3d).

**Fig. 4:**
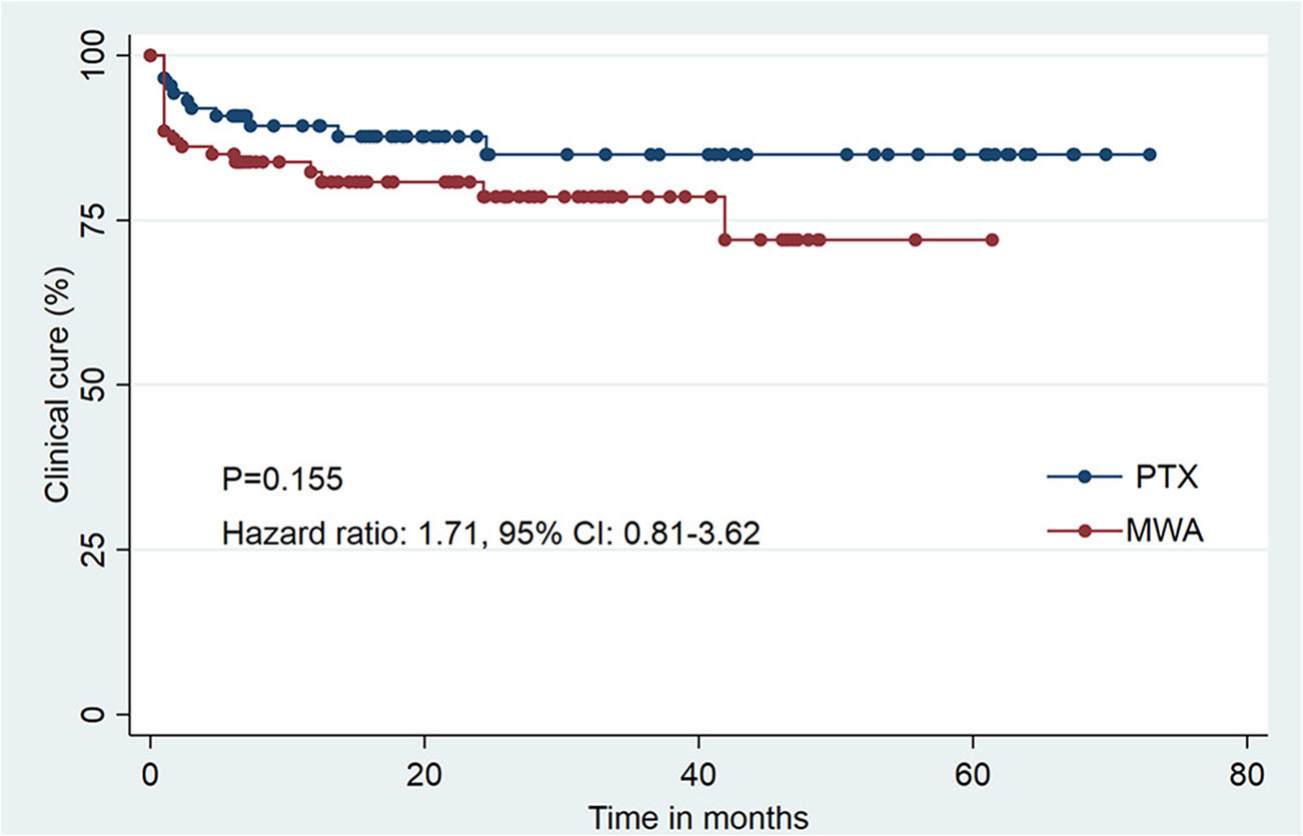
Graphs show Kaplan-Meier survival estimates for clinical cure between propensity score–matched patients who underwent microwave ablation (MWA) or parathyroidectomy (PTX)

The median follow-up was 22.5 months (IQR, 6.1–48.5 months) in the MWA group and 21.0 months (IQR, 6.0–66.1 months) in the PTX group (*p* = .546). In the MWA group, the clinical cure rates at 6 months, 1 year, 3 years, and 5 years were 85.1%, 82.3%, 78.5%, and 72.0%, respectively. In the PTX group, the clinical cure rates at the same follow-up time were 90.8%, 89.3%, 85.0%, and 85.0% (Table 3). There was no significant difference in the clinical cure rates between the two groups (hazard ratio, 1.71, 95% CI: 0.81–3.62; *p* = .155, Fig. 4). The rates of persistent and recurrent pHPT were also not significantly different between the two groups (recurrent pHPT rate: MWA 13.8% vs. PTX 10.3%, *p* = .643; persistent pHPT rate: MWA 6.9% vs. PTX 3.4%, *p* = .496).

**Table 3:** Comparison of clinical cure rate in patient with pHPT between the MWA and PTX group

Follow-up time	Clinical cure rate (%)		*p*
	MWA	PTX	
6 month	85.1 (75.7–91.0)	90.8 (82.5–95.3)	0.204
1 year	82.3 (77.4–89.0)	89.3 (80.4–94.3)	0.171
2 year	80.8 (70.5–87.8)	85.0 (73.7–91.7)	0.317
3 year	78.5 (67.4–86.3)	85.0 (73.7–91.7)	0.243
4 year	72.0 (53.9–84.0)	85.0 (73.7–91.7)	0.135
5 year	72.0 (53.9–84.0)	85.0 (73.7–91.7)	0.135

Data in parentheses is 95% confidence intervals

*MWA* microwave ablation, *PTX* parathyroidectomy

A total of 21 patients underwent operative failure: 12 (13.8%, 12/87) in the MWA group and 9 (10.3%, 9/87) in the PTX group. There was no difference in the operative failure rate between the two groups (*p* = .643). The levels of serum iPTH and calcium increased in 8 patients (MWA: PTX = 5:3), while in the other 13 patients (MWA: PTX = 7:6), only serum iPTH increased.

### Risk factors associated with persistent and recurrent pHPT

Multiple clinical parameters were analyzed for screening out the risk factors associated with persistent or recurrent pHPT. The statistical results showed that a high level of preoperative iPTH (hazard ratio, 1.00, 95% CI: 1.00–1.00, *p* = .012) and multiple pHPT nodules (hazard ratio, 3.56, 95% CI: 1.78–7.09, *p* < .001) were independent prognostic factors of recurrent and persistent pHPT in the two groups. However, the treatment modality was not a risk factor that contributed to recurrent or persistent pHPT (hazard ratio, 1.7, 95% CI: 0.8–3.6; *p* = .16) (Table 4).

**Table 4:** Univariable and multivariable analyses of risk factors for recurrent and persistence pHPT in the matched cohort

Parameter	Univariable analysis		Multivariable analysis	
	Hazard ratio	*p*	Hazard ratio	*p*
Sex (male vs female)	0.78 (0.37, 1.66)	0.519	–	
Age (years)	1.01 (0.99, 1.04)	0.328	–	
BMI (kg/m^2^)	1.11 (1.00, 1.23)	0.051	1.10 (0.98, 1.25)	0.118
Symptomatic PHPT (yes vs. no)	1.54 (0.72, 3.32)	0.268	–	
Serum iPTH (pg/mL)	1.00 (1.00, 1.00)	0.175	1.00 (1.00, 1.00)	0.012
Serum calcium (mmol/L)	0.48 (0.12, 1.82)	0.278	–	
Serum phosphorus (mmol/L)	0.37 (0.05, 2.74)	0.328	–	
ALP (U/L)	0.99 (0.99, 1.00)	0.197	0.99 (0.98, 1.00)	0.056
Vitamin D (nmol/L)	0.99 (0.97, 1.02)	0.585	–	
eGFR (mL/min/1.73m^2^)	0.99 (0.97, 1.00)	0.166	1.00 (0.98, 1.03)	0.905
Creatinine (umol/L)	1.01 (1.00, 1.02)	0.087	1.00 (0.99, 1.03)	0.538
Estimated blood loss (mL)	0.97 (0.91, 1.03)	0.273	–	
Procedure time (minute)	0.99 (0.98, 1.00)	0.122	0.99 (0.96, 1.01)	0.346
Treatment modality (MWA vs PTX)	1.71 (0.81, 3.62)	0.162	0.71 (0.15, 3.26)	0.656
Major complications (yes vs. no)	1.94 (0.59, 6.41)	0.278	–	
Nodule diameter (cm)	1.19 (0.79, 1.79)	0.405	–	
Nodule volume (mL)	1.05 (0.94, 1.16)	0.404	–	
No. of nodule	3.58 (1.93, 6.62)	< 0.001	3.56 (1.78, 7.09)	< 0.001
Nodule location (superior vs. inferior)	1.03 (0.48, 2.22) 0.932	–		

Data in parentheses are 95% confidence intervals. The Cox proportional hazards regression model was used for the univariable and multivariable analysis. Variables with *p* < 0.20 in univariable analyses were included in the multivariable model

*eGFR* estimated glomerular filtration rate, *PTX* parathyroidectomy, *ALP* alkaline phosphatase, *BMI* body mass index

### Complications

Voice change, as a major complication, was observed in 6 patients (6.9%) in the MWA group and 2 patients (2.3%) in the PTX group. Six patients’ voices recovered completely within 9 months in the two groups (MWA: 4.6%; PTX: 2.3%). Two (2/87, 2.3%) patients experienced permanent nerve paralysis that resulted in hoarseness throughout the follow-up period in the MWA group. In the PTX group, major complications—dyspnea caused by hematoma—occurred in one patient and were relieved after surgical hemostasis. There was no significant difference in the rate of major complications between the MWA and PTX groups (6.9% vs. 3.4%, *p* = .496). The incidences of transient hypoparathyroidism (MWA vs. PTX, 33.3% vs. 62.1%, *p* < .001) and hypocalcemia (MWA vs. PTX, 4.6% vs. 18.4%, *p* = .008) were higher in the PTX group. Regarding the mean duration of transient hypoparathyroidism or transient hypocalcemia, there was no difference between the two groups (Table 2).

## Discussion

Primary hyperparathyroidism is most commonly due to a single benign parathyroid adenoma (80%) [1, 2]. Parathyroidectomy is the definitive treatment for pHPT as recommended by guidelines [5, 7]. MWA is a rapidly developing minimally invasive technique that has shown advantages in the treatment of pHPT [12, 13, 15, 16, 18]. Given the high thermal efficiency of MWA and efficient hydrodissection, the safety and effectiveness of MWA have been improved [19, 20].

In a previous study based on 56 patients with pHPT who were followed up for a 6-month period after treatment, Liu et al showed that MWA and PTX provided comparable results in terms of cure rate (MWA vs. PTX: 82.1% vs. 89.3%) and complications (MWA vs. PTX: 21.4% vs. 25%) [16]. Our study, based on a larger sample size (174 patients) and a longer follow-up period (28.5 months), obtained similar results, confirming a comparable efficacy of MWA and PTX. Furthermore, the MWA group had a shorter procedure time than those in the PTX group; the incision was only one 1-mm pinhole, and the procedure could be performed under local anesthesia. Especially for selected patients, such as those with hypercalcemic crisis, senile patients with comorbidities who were unfit for surgery and young female patients who were anxious about scarring, MWA could be considered an alternative treatment. In addition, in the present study, the hospitalization time was 6 days in the MWA group, as patients were generally hospitalized for timely detection and management of delayed hematoma and, if necessary, for comprehensive treatment such as calcium supplementation and medical insurance reimbursement.

In terms of major complications, there was also no significant difference between the MWA and PTX groups (6.9% vs. 3.4%), while two patients (2.3%) experienced permanent hoarseness during the follow-up period in the MWA group. Although the incidence of permanent hoarseness was higher in the MWA group, the difference was not statistically significant. In previous studies, the incidence of permanent nerve paralysis after MWA was lower than that after PTX (2.3% vs. 3.9%) [21].

The multivariable analyses showed that high levels of preoperative iPTH and multiple pHPT nodules were independent risk factors for recurrent and persistent pHPT. This result was consistent with the findings of previous studies [22, 23]. Several retrospective series have shown that concordant imaging of US and MIBI was accurate in 95–100% of patients with single-gland disease; however, the same was not true for multiglandular disease [24–26]. Some researchers have suggested that iOPTH monitoring might be a helpful but imperfect adjunct [27, 28]. Multiglandular disease, especially some ectopic or small pHPT nodules, increased the difficulty and challenge for both MWA and PTX. Therefore, patients with multiple pHPT nodules might warrant more rigorous intraoperative scrutiny and more vigilant monitoring after treatment.

As the traditional treatment of pHPT, PTX possesses definite curative effectiveness and has its own advantages: (1) pHPT nodules are completely removed, and pathological results can be obtained. (2) Large pHPT nodules can be easily removed in one procedure. (3) Ectopic pHPT nodules (posterior sternum, mediastinum) can be managed. However, PTX is more invasive and causes scarring. Some senile patients with comorbidities cannot tolerate general anesthesia. MWA is a new, promising therapeutic option for patients with pHPT with the following advantages: (1) Minimally invasive ablation is a relatively simple procedure under local anesthesia. (2) MWA has broad indications and is well tolerated by patients. (3) MWA requires a shorter procedure time and convalescence period. (4) MWA has good repeatability. Nevertheless, MWA also has a few limitations: preablation biopsy is generally waived because it cannot be used to make a differential diagnosis between benign and malignant pHPT nodules [5, 7]; some ectopic nodules cannot be detected by US; and larger nodules require fractional treatment.

There are a few limitations in the present study. First, given its retrospective nature, selection bias could not be excluded. Patients who receive MWA treatment might have pHPT lesions that are easily detected and accessible, and further randomized controlled trials should be conducted to reduce selection bias. Second, FNA of lesions candidate to MWA was not performed in the present study. Hence, one could argue that, while the histological diagnosis of parathyroid adenoma was always available after surgery, malignancy could not be excluded in patients submitted to MWA. It should however be underlined that while FNA of parathyroid lesions may be useful to confirm the localization of a parathyroid adenoma in challenging cases, cytology cannot reliably discriminate parathyroid carcinoma (quite rare) from adenomatous or hyperplastic lesions.

In conclusion, MWA and PTX are comparable in terms of efficacy and complications for the treatment of pHPT. MWA is minimally invasive and has a shorter procedure time. Multiple nodules and high levels of serum iPTH were risk factors for recurrent and persistent pHPT.

## Abbreviations

ALP: Alkaline phosphatase
MWA: Microwave ablation
PTH: Parathyroid hormone
PTX: Parathyroidectomy
pHPT: Primary hyperparathyroidism
RLN: Recurrent laryngeal nerve
US: Ultrasound

## Declarations

### Guarantor

The scientific guarantor of this publication is corresponding author Ming-an Yu

### Statistics and biometry

No complex statistical methods were necessary for this paper.

### Methodology

• retrospective

• observational

• performed at one institution
